# A Review of Tertiary Referrals for Management of Pediatric Esophageal Eosinophilia

**DOI:** 10.3389/fped.2018.00173

**Published:** 2018-06-20

**Authors:** Bridget Godwin, Chris Liacouras, Vijay Mehta, Joshua Eisenberg, Atu Agawu, Terri Brown-Whitehorn, Melanie A. Ruffner, Ritu Verma, Antonella Cianferoni, Jonathan M. Spergel, Amanda B. Muir

**Affiliations:** ^1^Division of Gastroenterology, Hepatology and Nutrition, Children's Hospital of Philadelphia, Philadelphia, PA, United States; ^2^Department of Pediatrics, Children's Hospital of Philadelphia, Philadelphia, PA, United States; ^3^Pediatrics, Cooper Medical School of Rowan University, Camden, NJ, United States; ^4^Division of Allergy and Immunology, Children's Hospital of Philadelphia, Philadelphia, PA, United States

**Keywords:** Eosinophilic esophagitis, proton pump inhibitor-responsive esophageal eosinophilia, guidelines, proton pump inhibitor, esophageal eosinophilia

## Abstract

**Background:** Eosinophilic esophagitis is a chronic, immune-mediated disease characterized by symptoms of esophageal dysfunction and ≥15 eosinophils/high-powered field (eos/hpf). Proton pump inhibitor responsive esophageal eosinophilia (1) is an entity of esophageal eosinophilia that responds to PPI therapy and is thought to be clinically and histologically similar to EoE. Current guidelines suggest therapy with PPI prior to endoscopy and use of PPI as first line for esophageal eosinophilia. In order to gain a better understanding of community practice patterns and to try differentiate between these two entities, we sought to evaluate the clinical presentations, treatment and final diagnoses of patients presenting to our institution for second opinions of esophageal eosinophilia.

**Methods:** A search of our electronic medical record yielded a list of patients presenting for a second opinion of esophageal eosinophilia. Charts were reviewed for clinical information.

**Results:** A total of 187 charts were included. Patients ranged from 1-19 years old with 75% being male and 74% being Caucasian. Of the patients who had documentation of their medications at the time of initial endoscopy, 70% were not on any PPI prior to their endoscopy, and 94% were on <2 mg/kg/day. Of the 19 patients who had full response to PPI therapy and were diagnosed with PPI-REE, close to half had previously been treated with diet, steroids, or both. Patients with final diagnosis of EoE had significantly higher eos/hpf on initial endoscopy compared to those with diagnosis of PPI-REE (51.9 ± 30.6 v. 35.8 ± 16.4. *p* = 0.027), as well as higher likelihood of having IgE-mediated food allergy (79 v. 47%, *p* = 0.003).

**Conclusions:** Diagnostic and therapeutic algorithms are needed for esophageal eosinophilia to prevent misdiagnosis and unnecessary procedures and therapies.

## Introduction

Eosinophilic Esophagitis (EoE) is a chronic, antigen and immune-mediated esophageal disease characterized by symptoms of esophageal dysfunction and ≥15 eosinophils/high-powered field (eos/hpf) isolated to the esophagus (2). The importance of treating EoE in children lies in both the effects symptoms have on development, nutrition, quality of life and feeding behaviors (3, 4), and the likelihood that EoE is a progressive disease leading to esophageal stricture (5). Proton pump inhibitor responsive esophageal eosinophilia (PPI-REE) (1) is an type of esophageal eosinophilia that responds to PPI therapy alone and is thought to be clinically, transcriptionally and histologically similar to EoE (1, 6, 7). The 2011 EoE guidelines suggest therapy with PPI prior to endoscopy and use of PPI as first line for esophageal eosinophilia (8). The diagnosis of of EoE is only made after endoscopy on the recommended 20–40 mg once or twice a day for 8–12 weeks in adults, and 1 mg/kg/dose, twice daily for 8–12 weeks in children (8).

The distinction between the diagnosis of PPI-REE and EoE is important as patients who respond to PPI for treatment of their esophageal eosinophilia can be treated with PPI as mono-therapy. Currently there is no therapy for EoE and patients are subjected to rigorous elimination diets or topical steroid preparations. There is evidence that 50.5% of adult patients with esophageal eosinophilia improve on PPI therapy (9) and up to 68.6% of children are PPI responsive (10). Furthermore, the majority of these patients with PPI-responsive esophageal eosinophilia are able to maintain remission upon weaning PPI to 40 mg daily or below (11). Therefore, appropriate PPI therapy may eliminate the need of lifelong dietary elimination or steroid therapy in patients with esophageal eosinophilia. High dose PPI therapy (2 mg/kg/day) alone is an important step in the diagnostic algorithm and can spare patients unnecessary therapies and procedures.

Recent adult studies showed a wide variety in diagnosis of and treatment for esophageal eosinophilia in adults (12, 13), with many being diagnosed with EoE and treated with dietary elimination or steroids prior to use of PPI. This trend has not yet been evaluated in the pediatric population. We sought to evaluate the clinical presentations, treatment and final diagnoses for patients presenting to our institution for second opinions of esophageal eosinophilia over the past 5 years. We also aimed to identify similarities and differences between patients with EoE and PPI-REE.

## Methods

### Study population

An IRB-approved chart review was done on all patients who presented to the Center for Pediatric Eosinophilic Disorders (CPED) at The Children's Hospital of Philadelphia (CHOP), for a second opinion for EoE between January, 2011 and February, 2017. This subspecialty clinic, which includes 3 care locations, is the largest provider of subspecialty allergy care services to patients residing in the eastern Pennsylvania, New Jersey, and Delaware region. In addition, we are an international referral center for EoE and have developed one of the largest pediatric cohorts for this condition.

The CPED program sees patients with a broad range of diagnoses in addition to EoE, including, but not limited to, hypereosinophilic syndrome and eosinophilic gastrointestinal disease (EGID). Charts were abstracted and determined for eligibility. For the purpose of this study, patients were excluded if they presented with a diagnosis other than EoE as an explanation of their esophageal eosinophilia, such as EGID or Crohn's Disease, if they never had >15 eos/hpf on esophageal biopsies, or if there were not enough records available for review. Patients identified as EGID were excluded from this review based on the presence of clinically significant eosinophilia outside of the esophagus per features outlined by Margaret Collins in 2014 (14). Included charts were reviewed by three pediatric residents, overseen by a fellow in Gastroenterology, as well as an attending physician. Any discrepancies or questions were brought to the attending physician for discussion and clarification. The charts were abstracted for clinical information, including symptom history, growth curves, medications, allergies, dietary therapies, endoscopic findings, pathology reports, treatments and diagnoses.

Patients were given a final diagnosis based on histologic findings on endoscopic biopsies as well as clinical course. Patients were defined as having esophageal eosinophilia if they had ≥15 eos/hpf on esophageal biopsies. If patients continued to have esophageal eosinophilia after 8 weeks on a high-dose PPI, they were given the diagnosis of EoE. If esophageal eosinophilia resolved with initiation or increase of PPI patients were given the final diagnosis of PPI-REE.

Diagnosis of IgE-mediated food allergy and EoE was made in accordance with established practice parameters (8, 15). Evidence of IgE-mediated allergy was defined as the presence of consistent history and laboratory analysis, skin testing, and/or food challenge.

Statistical analysis was performed on histologic and clinical data collected on all patients included in the study as noted above. Chi-squared analysis was used for comparison of proportions. Two-sample *t*-test was used to compare continuous variables. An α of 0.05 was used for significance.

## Results

### Patient characteristics

**Figure 1:**
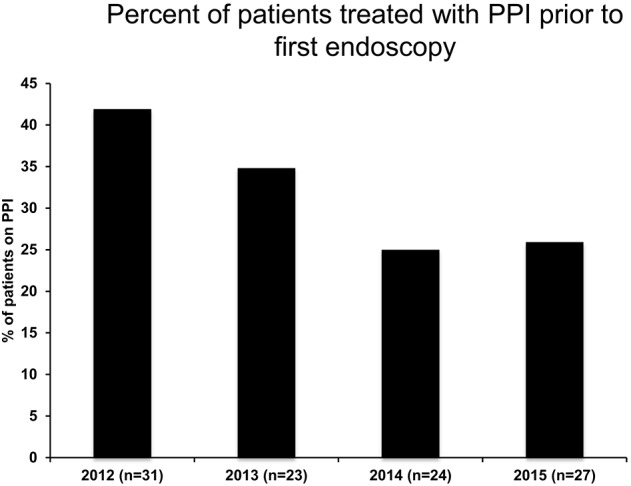
PPI administration rate prior to first endoscopy following publication of 2011 EoE Guidelines.

We identified 187 patients diagnosed with EoE refered for second opinion, 3 of which were referred internally from our own institution (Table 1, Figure 1). The patients ranged from 1 to 19 years of age. Indications for initial endoscopy included vomiting and regurgitation in 104 (55.6%), dysphagia in 76 (40.4%), food impaction in 19 (9.6%) and failure to thrive (FTT) in 33 (17.6%).

**Table 1:** Main demographic and clinical features of the study population.

**DEMOGRAPHICS (*n* = 187)**	
Age (mean)	8.6 years
Male gender	141 (75%)
Caucasian	138 (74%)
**ATOPIC FEATURES**	
Asthma	90 (48%)
Atopic dermatitis	76 (41%)
Allergic rhinitis	132 (71%)
IgE-mediated food allergy	139 (74%)
**CLINICAL FEATURES**	
History of stricture^†^	6 (3.2%)
History of weight <5th %ile	30 (16%)
History of food impaction^‡^	23 (12.3%)
History of dysphagia	76 (40.6%)

† *Based on imaging or endoscopy*.

‡ *Food, coin, or pill impaction*.

### PPI-trial prior to diagnosis of EoE is uncommon

The current guidelines (8) state that in order to diagnose EoE, pediatric patients must receive a PPI trial with 2 mg/kg/day for 6–8 weeks prior to their endoscopy. In looking at prescribing trends, we observed that in the years following the guideline publication, there was an overall decrease in following the guidelines and using PPI- at any dose prior to endoscopy over time with 41.9% of patients having some PPI exposure prior to their first endoscopy in the year 2012, but only 25.9% of patients having PPI trial by 2015 (Figure 1).

**Figure 2:**
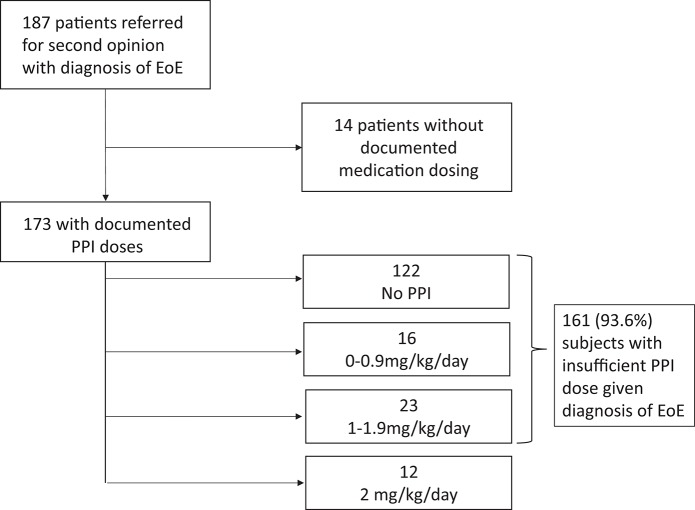
PPI Doses at Time of Initial Endoscopy.

Data analysis further revealed that of 173 patients who had documentation of medications at the time of EoE diagnosis, 161 (93.6%) were on <2 mg/kg/day of PPI. 122 (70%) of these patients were on no PPI at all (Figure 2). Only 12 patients (6%) were on 2 mg/kg/day of PPI prior to their first endoscopy.

### Steroids and diet elimination are commonly used prior to PPI trial

**Figure 3:**
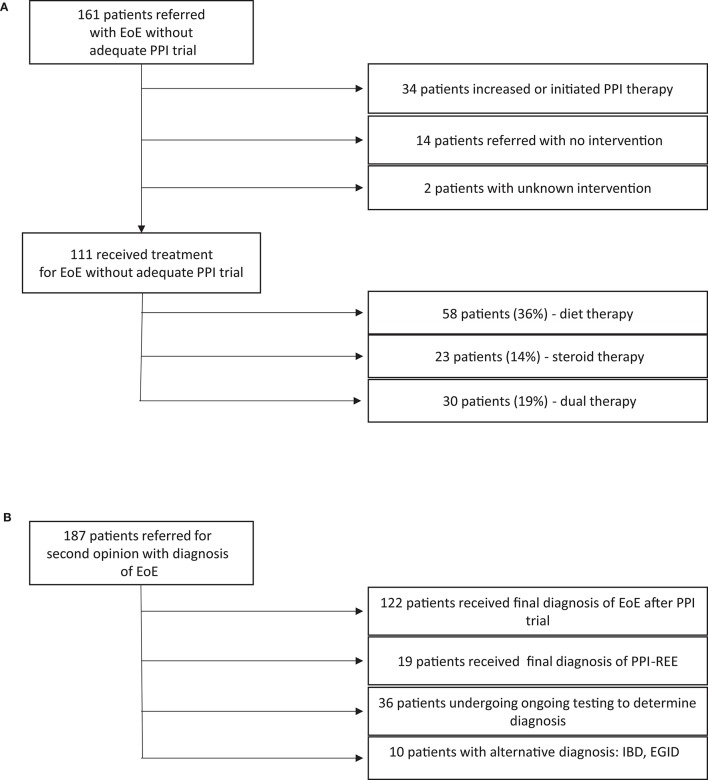
**(A)** Therapies attempted in patients with esophageal eosinophilia. **(B)** Final diagnoses given after further workup.

Of 161 patients diagnosed with EoE despite inadequate PPI therapy, 111 began therapy for EoE with dietary elimination (36%), corticosteroids (14%) or both (19%) without first being trialed on high dose PPI alone (Figure 3A). Of the 50 patients not treated with dietary elimination or corticosteroids, 34 were started on PPI or had an increase in their dose of PPI without initiation of other therapy, and 14 were referred for a second opinion without intervention. Intervention was unable to be extracted from the chart for two of the patients.

### Change in diagnosis is common after PPI trial

Of 187 patients included in our study, 19 (10.2%) had a final diagnosis with PPI-REE after evaluation/treatment at CHOP. 122 patients (65%) maintained their diagnosis of EoE, 10 (5.3%) were diagnosed with another disorder (EoG, IBD, etc.) and 36 (19.3%) did not have a final diagnosis at time of analysis (Figure 3B).

### Dietary restriction and numerous EGDs common in PPI-REE patients

Of the 19 patients with the final diagnosis of PPI-REE, 16 of them (84%) were not on any PPI at the time of initial endoscopy. Eight of the 19 patients had persistent eosinophilia on a PPI that resolved with increase in the PPI. The average PPI dose at which eosinophilia resolved was 1.5 mg/kg/day. Patients with final diagnosis of PPI-REE had up to 13 foods eliminated from their diet, and had an average of 2.1 endoscopies before initiation of high dose PPI, though this value ranged from 1 to 7 (Table 2). Two patients had been treated with swallowed steroids in addition to dietary elimination. Follow-up information was available on 10 of the patients diagnosed with PPI-REE. Of these 10, 2 had been weaned off of PPI at the time of data analysis. The others remained on high dose PPI.

**Table 2:** Characteristics of patients with final diagnosis of PPI-REE.

**Patient**	**Foods eliminated prior to initiation of high dose PPI^†^**	**No. of endoscopies prior to high dose PPI**	**PPI dose (mg/kg/day) at initial endoscopy**	**PPI dose with persistent eosinophilia (mg/kg/day)**	**PPI dose at which eosinophilia resolved (mg/kg/day)**
1	Soy, nuts	7	0	0.6	1.3
2	Elemental diet	1	0	N/A	1.9
3	None	1	0	N/A	1.4
4	Milk, egg, soy, wheat, peanut, tree nut, fish, shellfish	4	0	N/A	0.6
5	None	1	0	0.8	1.5
6	Soy,nuts, carrot, corn	3	0	0.3	0.64
7	None	2	Unknown		2
8	Milk	1	1.6	1.4	2.1
9	None	1	0	N/A	1.8
10	None	1	0	0.4	1.7
11	None	1	0	0.3	0.6
12	None	1	0.8	0.8	2.
13	None	1	0	N/A	1.7
14	None	1	0	N/A	0.9
15	Milk	3	0.8 (only 1 week prior)	0.7	1.5
16	Soy	2	0	N/A	1.7
17	None	3	0	N/A	2.4
18	Wheat, soy, sesame, walnut, peanut, carrot, milk, tomato, egg, blueberries, strawberries, fish and shellfish	4	0	0.6	1
19	Milk, avocado	2	0	0.91	1.6

† *Foods eliminated due to IgE mediated allergy not included*.

### Differences exist between patients with EoE and PPI-REE

Previous reports have failed to show any clinical and endoscopic differences between EoE and PPI-REE (16). Indeed we found that age at presentation, gender and ethnicity did not vary between the groups. However, PPI-REE patients were more likely to have a history of “regurgitation” or “abdominal pain” as their presenting symptom than patients with the final diagnosis of EoE. We also found that patients with a final diagnosis of EoE had significantly higher eos/hpf found on initial endoscopy compared to those with final diagnosis of PPI-REE (51.9 ± 30.6 vs. 35.8 ± 16.4, *p* = 0.027), as well as a higher likelihood of having IgE-mediated food allergy (79 vs. 47%, *p* = 0.003). There was no difference in prevalence of atopic dermatitis, asthma, or allergic rhinitis between the groups (Table 3).

**Table 3:** Differences between patients with PPI-REE and EoE.

	**PPI-REE (*n* = 19)**	**EoE (*n* = 122)**	*p*-value^*^
Age at initial presentation, (mean ± st. dev)	5.3 ±4.3	4.8 ± 4.6	0.67
Male Gender, n (%)	12 (63)	99 (81)	0.08
White ethnicity, n (%)	11 (58)	93 (76)	0.099
Eos/hpf at initial presentation, (mean ± st. dev)	35.8 ±16.4	51.9 ± 30.6	**0.027**
Hx of IgE-mediated food allergy, n (%)	9 (47)	96 (79)	**0.003**
Hx of asthma	8 (42)	59 (48.4)	0.605
Hx of atopic Dermatitis	7 (37)	52 (43)	0.635
Hx of allergic Rhinitis	11 (58)	87 (71)	0.24
Hx of dysphagia, n (%)	10 (53)	63 (52)	0.94
Hx of vomiting, n (%)	10 (53)	63 (52)	0.94
Hx of chest or throat pain, n (%)	1 (5.3)	17 (14)	0.29
Hx of heartburn, n (%)	10 (53)	41 (34)	0.11
Hx of regurgitation, n (%)	6 (32)	14 (12)	**0.02**
Hx of abdominal pain, n (%)	13 (68)	46 (38)	**0.014**
Hx of failure to thrive, n (%)	3 (16)	24 (19.7)	0.69
Hx of food impaction^†^, n (%)	1 (5.3)	16 (13.1)	0.33
Hx of stricture^‡^, n (%)	1 (5.3)	5 (4.1)	0.81

* *Chi squared analysis*.

† *Food, coin or pill*.

‡ *Based on imaging or endoscopy*.

*Bold values signifies a p-value of <0.05*.

## Discussion

Our study highlights not only that practice varies in the community but that this variation can lead to delay in diagnosis as well as unnecessary treatment with dietary elimination and use of steroids in patients who have PPI-responsive esophageal eosinophilia. There is great discussion in the field at this time regarding the overlap between EoE and PPI-REE, as well as the likelihood that they are not separate entities (1, 17). As this discussion takes place, this paper is crucial to highlighting the concrete effects on patients that do not undergo initial PPI trial. For this reason we have chosen to use the terminology that continues to distinguish between these two entities.

Strengths of this study include the use of a large population of patients with esophageal eosinophilia referred to a tertiary care center. It offers a novel look at how pediatric gastroenterologists are diagnosing and treating EoE, and describes a population of EoE patients that is demographically similar to the broader EoE population in terms of race and gender. A possible limitation of our study is that it specifically evaluated a pool of patients presenting for second opinions, therefore selecting for patients who were more likely to have difficult to control esophageal eosinophilia. However, this is also a strength as it highlights difficult cases that are less likely to have been put on PPI prior to endoscopy and therefore able to be captured for a study such as this. Another potential weakness of using a tertiary care referral center as a basis for our study is that not all of the pediatric gastroenterologists in the community were included, only those that had patients who were referred. For this reason, our study may not fully describe community-prescribing practices. Additionally, providers were not asked about their reasoning behind deviating from guidelines and therefore it is not possible for us to discern if their practices were due to non-adherence or non-acceptance of clinical guidelines. A survey of all community providers would be valuable as a future study.

In the field of esophageal eosinophilia there is discussion of eliminating the need for PPI trial prior to initial endoscopy when evaluating for esophageal pathology (1). This is being proposed in order to avoid missing diagnoses of PPI-REE in patients that undergo endoscopy already on high dose PPI. There are guidelines that have been published since the completion of this study (17) which suggest endoscopy prior to PPI trial in patients displaying symptoms consistent with EoE. There is currently low evidence for this approach but strong expert support. While a potential limitation of this study is that the algorithm that it is focused on is being called into question, this particular controversy makes this study all the more relevant. The strategy laid out in the 2017 guidelines (17) may be prudent in adults who seem to exhibit more predictable symptoms such as dysphagia and recurrent food impaction. Children, on the other hand, are more likely to display vague symptoms including failure to thrive, heart burn, abdominal pain, vomiting, and feeding refusal. It is difficult to justify an endoscopy as a first line approach in all children presenting to clinic with these symptoms without trying a PPI first especially given the risks of anesthesia in young children (18). It is also difficult to subject a young child to dietary elimination, which can be life-altering, and chronic use of swallowed steroids, before first seeing if they respond to PPI therapy. This is a dilemma that requires complex decision-making and weighing the risks and benefits of increased anesthesia time vs. diagnostic accuracy in the pediatric population.

While only 10% of patients included in this study had their diagnoses changed from EoE to PPI-REE, the burden of dietary elimination and re-introduction that these patients underwent, many over the course of years without symptomatic improvement, may outweigh the burden of a 8 week PPI trial. There are proposed risks of PPI therapy, such as kidney disease, bone fractures, and small intestinal bacterial overgrowth, however, a recent review has found that the quality of the evidence that suggest these adverse effects is low or very low (19). A 2018 pediatric study found that up to 70% of children with PPI-REE remained in remission on low-dose PPI, and the PPIs were found to have an acceptable safety profile (20). In fact, our data show that an average dose of 1.5 m/kg/day was enough to resolve eosinophilia in patients with PPI-REE. While attempts were made at our institution to consistently dose patients as close to 2 mg/kg/day as possible, due to challenges in dosage formulations, with capsules and packets being distributed in 5 mg aliquots, this was not always possible. As a result, we are able to report truly administered dosages in a practice where close adherence to the 2011 guidelines was attempted.

Of note, it is likely that we are underestimating the number of patients included in this study diagnosed with PPI-REE as final diagnoses were not available for many of our patients due to the fact that they presented with numerous foods eliminated from their diet before being trialed on a PPI, necessitating introduction of foods in a step-wise manner. Many of these patients are currently undergoing systematic reintroduction of foods, which may involve multiple scopes and in some cases day-hospital food trials. In these cases the burden of not doing a PPI trial is great.

Our study highlights the similarities and differences between the patients with final diagnoses of EoE vs. PPI-REE. While it has been recently postulated that EoE and PPI-REE are the same entity (21), our study did identify distinctions between the two populations. While severe clinical findings such as history of dysphagia or food impaction did not significantly differ between the two groups, this may be a consequence of this being a pediatric study and these symptoms occur more frequently in adults. Abdominal pain and regurgitation on the other hand, two symptoms that are not specific to EoE, had higher prevalence in the PPI-REE population than the EoE population. There were also differences in clinical features, including eos/hpf found on initial endoscopy and history of IgE-mediated food allergy. Our specific finding of increased eos/hpf in patients with the final diagnosis of EoE supports previous literature which showed that eosinophil count on initial EGD is higher in patients that do not respond to PPI (10, 22). These differences suggest that while there are similarities between the two entities, there may be distinctions to distinguish one from the other. This type of data collection may be used in future prospective population studies to identify clinical differences between PPI-REE and EoE, eventually allowing for distinction between the two populations without need for a PPI trial.

Although there is emerging evidence that patients with PPI-REE may also respond to dietary therapy (23), there are consequences of dietary elimination and food restriction, both nutritional as well as in regard to quality of life (24). Many patients with the final diagnosis of PPI-REE in this study did not in fact respond to dietary therapy, and in those cases dietary trial prolonged their recovery. This delay in treatment likely also has negative consequences, given that symptomatic EoE can lead to behavioral changes such as food refusal (3). Our current study highlights that the 2011 guidelines, while currently controversial, were not being followed even in the years preceding the current controversy, suggesting that the implementation of the guidelines in practitioners was inconsistent. While this may reflect a gap in knowledge amongst practitioners, it may also reflect a need for improvement in the way the groups writing guidelines communicate to the target audience in a way that is accepted. Our study shows the importance of a standard that is able to be followed, as the current practice lead to unnecessary dietary elimination, steroids, and numerable endoscopies in the patients we described.

## Ethics statement

As per the Frontiers authors guidelines, you are required to use the following format for statements involving human subjects: This study was carried out in accordance with approved by the recommendations of Institutional Review Board at the Children's hospital of Philadelphia. In this protocol a waiver of consent was granted to due to the retrospective nature of the project.

## Author contributions

CL, AM, and BG: experimental design; BG, CL, VM, JE, AA, and AM: experimental execution; BG, CL, VM, JE, AA, MR, and AM: data analysis and interpretation; BG, CL, JE, AA, TB-W, MR, RV, AC, JS, and AM: manuscript writing/editing.

### Conflict of interest statement

The authors declare that the research was conducted in the absence of any commercial or financial relationships that could be construed as a potential conflict of interest.

## Abbreviations

CHOP: Children's Hospital of Philadelphia
EGD: esophagogastroduodenoscopy
EGID: eosinophilic gastrointestinal disorders
EoE: eosinophilic esophagitis
eos/hpf: eosinophils per high-powered field
FTT: failure to thrive
GERD: gastro-esophageal reflux disease
GI: gastrointestinal
H&E: Hematoxylin and Eosin
PEES: Pediatric Eosinophilic Esophagitis Symptom Scores; (1), proton pump inhibitor; (1), Proton pump inhibitor responsive esophageal eosinophilia.
